# Quality Evaluation of *Panax ginseng* Roots Using a Rapid Resolution LC-QTOF/MS-Based Metabolomics Approach

**DOI:** 10.3390/molecules181214849

**Published:** 2013-12-03

**Authors:** Dae-Young Lee, Jae Kwang Kim, Sabina Shrestha, Kyeong-Hwa Seo, Youn-Hyung Lee, Hyung-Jun Noh, Geum-Soog Kim, Yong-Bum Kim, Seung-Yu Kim, Nam-In Baek

**Affiliations:** 1 Department of Herbal Crop Research, National Institute of Horticultural and Herbal Science, RDA, Eumseong 369-873, Korea; 2 Division of Life Sciences, College of Life Sciences and Bioengineering, Incheon National University, Incheon 406-772, Korea; 3 Department of Oriental Medicinal Materials & Processing and Department of Horticultural Biotechnology, Kyung Hee University, Yongin 446-701, Korea; 4 Technology Services Division, National Institute of Horticultural and Herbal Science, RDA, Suwon 440-706, Korea

**Keywords:** Korean ginseng, metabolomics, multivariate analysis, PCA, PLS-DA, RRLC-QTOF/MS

## Abstract

Korean ginseng (*Panax ginseng* C.A. Meyer) contains several types of ginsenosides, which are considered the major active medicinal components of ginseng. The types and quantities of ginsenosides found in ginseng may differ, depending on the location of cultivation, making it necessary to establish a reliable method for distinguishing cultivation locations of ginseng roots. *P. ginseng* roots produced in different regions of Korea, China, and Japan have been unintentionally confused in herbal markets owing to their complicated plant sources. PCA and PLS-DA using RRLC-QTOF/MS data was able to differentiate between ginsengs cultivated in Korea, China, and Japan. The chemical markers accountable for such variations were identified through a PCA loadings plot, tentatively identified by RRLC-QTOF/MS and partially verified by available reference standards. The classification result can be used to identify *P. ginseng* origin.

## 1. Introduction

Korean ginseng, the root of *Panax ginseng* C.A. Meyer, is a medicinal herb commonly used in traditional Korean medicine and is now widely used around the World [1]. Recent phytochemical and pharmacological studies have demonstrated that the extract and bioactive components of ginseng include saponins, polyacetylenes, sesquiterpenes, and polysaccharides [2,3]. However, the therapeutic effects of ginseng roots are primarily associated with triterpenoids and ginseng saponins [4]. Qualitative and quantitative properties of ginseng saponins, known as ginsenosides, may differ significantly, depending on the area of cultivation of the *P. ginseng* [5]. In addition, the efficacy and bioactive components of ginseng roots may differ depending on the cultivation region [6]. Accordingly, it is necessary to distinguish the cultivation region of ginseng roots and the components present. *P. ginseng*, cultivated in Korea, China, and Japan, has been unintentionally mislabeled and/or confused in Korean herbal markets due to its complicated plant sources. Therefore, it is necessary to establish a reliable technique to distinguish between these herbal drugs.

Recently, improvements in chromatographic performance have been achieved by the introduction of rapid-resolution liquid chromatography (RRLC), applying the technique of small particles (sub-2 µm) packed into short columns run at a relatively high flow rates [7]. This technique takes full advantage of chromatographic principles to achieve superior numbers of theoretical plates and perform very high-resolution separations in a short time with little solvent consumption [8]. The ongoing development of RRLC coupled to TOF-MS offers a new strategy to perform compositional and structural analyses of complex constituents in complicated mixtures. In addition to the rapid acquisition rates and large mass range detection, greater accuracy in mass measurements is also achieved by TOF-MS for both qualitative and quantitative analyses [9]. Moreover, the RRLC-TOF-MS method has absolute advantages over previous approaches in terms of sensitivity and selectivity. RRLC-TOF-MS can also be used to establish a fast and efficient analytical method for simultaneous determination of the cultivation area of ginseng, Korea, China, and Japan [10].

In this study, we used RRLC-TOF-MS to analyze secondary metabolites variations for the discrimination of various *Panax ginseng* roots through the application of metabolite fingerprinting. Principal component analysis (PCA) was employed to observe dissimilarities and elucidate how quality discrimination is resolved in correlation with influential factors. Good predictive partial least squares (PLS) models were also constructed. In addition, tentative identifications of some significant compounds from the separations were made, and a further understanding on how classification in ginseng could be expressed and correlated to quality was attained.

## 2. Results and Discussion

### 2.1. Investigation of RRLC-MS Conditions

**Figure 1:**
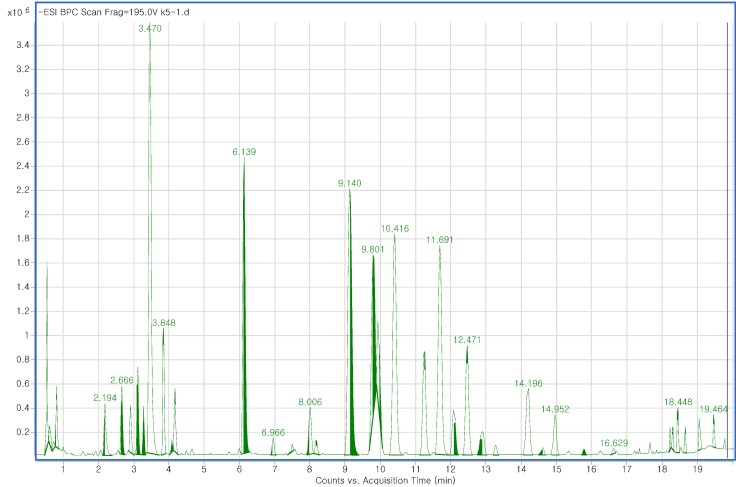
RRLC-QTOF/MS, ESI^−^ base peak intensity (BPI) chromatogram of *Panax ginseng* roots.

The fast chromatographic separation of ginsenoside was carried out by RRLC in order to avoid excessive system pressure. The most efficient way to attain rapid analysis time while also generating high column efficiency and resolution is by utilizing a small particle size column. Therefore, chromatographic analysis was executed using a 2.1 × 100 mm, 1.8 µm particle size RRHT C_18_ column. In a short time (20 min), higher sensitivity is achieved as higher resolution peaks with narrower widths are produced, making the RRLC analysis system more intensive. In the present study, LC-QTOF/MS spectra were recorded three times per sample. The high resolution and speed of RRLC combined with the accurate mass measurement of QTOF/MS allowed a total of 250 components, including 27 standard ginsenosides, to be simultaneously separated in 20 min. Among the solvent conditions, the *n*-BuOH solvent system, comprising *n*-BuOH extraction after partition with CHCl_3_ for MeOH extracts, and the mixture of CHCl_3_-MeOH-H_2_O as extraction solvent did not yield a good chromatogram. However, ginseng roots were extracted well when 70% MeOH was used. Different extraction temperatures (30 °C, 40 °C and 50 °C) were tested and among them, 50 °C resulted in the largest number of peaks and good separation with a slight reduction of peak signal. The column temperature was set at 45 °C to alleviate extra column pressure resulting from the higher flow rate, which can improve chromatographic separation and peak shape. Isocratic elution led to no obvious separation because of the similar polarities and structures of most ginsenosides, while gradient elution gave better separation. In addition, the effects created with the addition of formic acid to the eluting solvent at different concentrations (0.1%, 0.05% and 0.01%) were tested. We found that 0.1% formic acid gave the best separation with only a slight reduction of peak signal. Both positive and negative ion modes were tested for electrospray ionization (ESI) instruments. However, chromatographic data from the positive mode showed poor ionization stability. Therefore, multivariate analysis was carried out with data from the negative mode only. Accurate mass measurements were obtained by means of an automated calibration delivery system, which contains the internal reference masses at *m/z* 112.9856, 1033.9881, and 1933.9306 in the negative ion mode. Data were collected between 100 and 1,500 *m/z*. Each ginsenoside peak was identified in the total ion chromatogram by matching the molecular ions in the mass spectra with those in the standard samples and *in-house* library (Figure S1). The based peak intensity (BPI) chromatogram obtained in negative ion mode for a Korean ginseng root is shown in Figure 1.

### 2.2. PCA of Ginseng Root Samples

**Figure 2:**
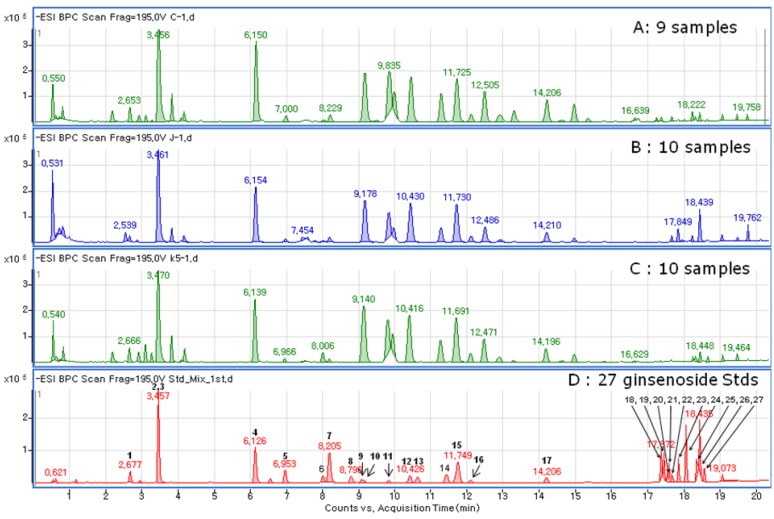
Chromatograms for three *Panax ginseng* root extracts including China (**A**, Cn), Japan (**B**, Jp), Korea (**C**, Kr), and standard of ginsenosides (**D**).

**Figure 3:**
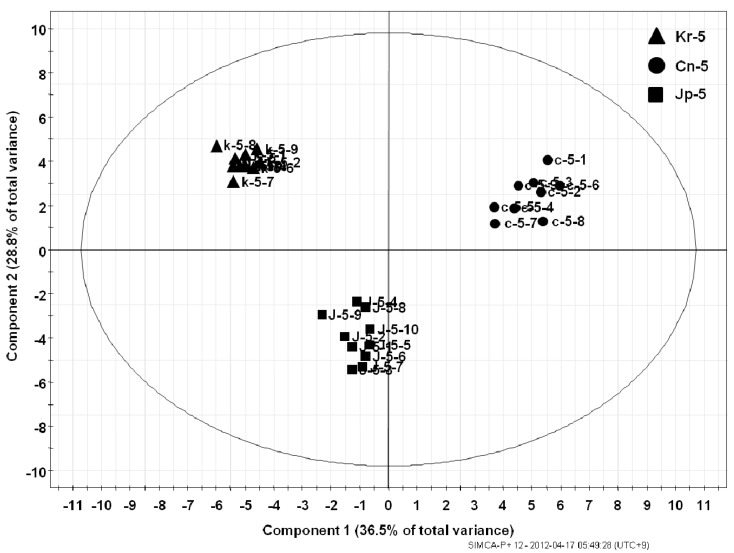
PCA score plot representing analysis derived from ESI negative ion mode. Separation is depicted by the PCA score plot revealing the significant influence of cultivation area (*i.e.*, Korea, China and Japan) in determining dissimilarities.

**Figure 4:**
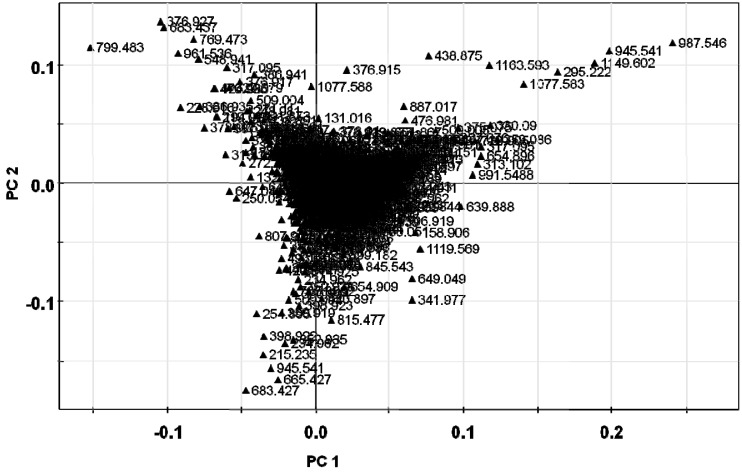
PCA loadings plot obtained using Pareto scaling with mean centering.

The metabolite fingerprinting of 5-year-old ginseng samples (Kr-, Cn-, and Jp-5) (Figure 2) was carried out using PCA, an unsupervised multivariate pattern recording method, in order to efficiently visualize the dissimilarities based on the chromatographic pattern [11]. Mean-centered and par-scaled (scaled to the square root of SD) mathematical methods were performed to pretreat the data sets resulting from the above samples obtained with the SIMCA-P^+^ 12.0 software [11]. The PCA results showed distinct separations among all three types of ginseng samples (Kr-, Cn-, and Jp-5) (Figure 3). The PCA score plots representing analyses derived from negative ionization mode described 65.3% of the total variance in which optimal segregation was achieved between PC 1 (36.5%) and PC 2 (28.8%), where PC 1 was the key component for sample separation. The Cn-5 and the others were clearly separated by the principal component 1 (PC1), whereas the Jp-5 was easily discriminated by the principal component 2 (PC2) with the others. The corresponding loading plot of the negative ion mode was consistent with its respective score plot, in which significant intensities of specific RT-*m/z* variables to their correlative clusters apparently implicated variations in the metabolite fingerprints (Figure 4). The preferential distribution of marker ions (*m/z* 799.4835: Rf, 683.4375: F1, 769.4731: R2, 961.5360: 20-glc-Rf, 376.927: unknown) in the first quadrant of the loadings plot accounted primarily for the difference of Korean *P. ginseng* (Kr-5). Analogously, the distribution in the second and third quadrants of the loading plots indicated the variations of Chinese (Cn-5; *m/z* 987.5467 [M−H]^−^, 1149.6025 [M−H]^−^, 1077.5835: Rc) and Japanese (Jp-5; *m/z* 683.4275: Rh1, 665.4270: Rk3, 945.5416: Rd) *P. ginseng*, respectively. The experimentally determined *m/z* of the selected marker ions was used to compute the possible calculated mass and mass accuracy (mDa and ppm). Elemental compositions associated with the measured mass of the marker ions were generated and studied using Mass Hunter Workstation Data acquisition software (B.03.00). Ginsenosides were verified by comparing the major fragment ions and retention times of marker ions with those of available standards and accurate mass measurement (Table 1).

**Table 1:** *In-house* library of ginsenosides isolated from *Panax ginseng* roots.

No.	R.T. (min)	Identification	Molecualr formula	Theoretical accutare mass [M]^+^	Calculated mass [M−H]^−^	QTOF/MS (ESI−) [M−H]^−^	Mass accuracy (ppm)
1	2.659	20-*O*-gluco-ginsenoside Rf	C_48_H_82_O_19_	962.5450	961.5372	961.5360	−1.2
2	3.431	ginsenoside Rg1	C_42_H_72_O_14_	800.4922	799.4844	845.4912 [M−H+HCOOH]^−^	1.3
3	3.435	ginsenoside Re	C_48_H_82_O_18_	946.5501	945.5423	945.5415	−0.8
4	6.109	ginsenoside Rf	C_42_H_72_O_14_	800.4922	799.4844	799.4835	−1.1
5	6.942	notoginsenoside R2	C_41_H_70_O_13_	770.4816	769.4738	769.4731	−0.9
6	8.002	ginsenoside Rg2	C_42_H_72_O_13_	784.4973	783.4894	783.4830	−8.1
7	8.154	ginsenoside Rh1	C_36_H_62_O_9_	638.4394	637.4315	683.4275 [M−H+HCOOH]^−^	5.9
8	8.747	ginsenoside Ra2	C_58_H_98_O_26_	1210.6346	1209.6268	1209.6303	2.9
9	9.087	ginsenoside Rb1	C_54_H_92_O_23_	1108.6029	1107.5951	1107.5886	5.8
10	9.107	ginsenoside Ra3	C_59_H_100_O_27_	1240.6452	1239.6373	1239.6312	−4.9
11	9.723	ginsenoside Ro	C_48_H_76_O_19_	956.4981	955.4902	955.4922	2.0
12	10.380	ginsenoside Rc	C_53_H_90_O_22_	1078.5923	1077.5845	1077.5835	−0.9
13	10.600	ginsenoside Ra1	C_58_H_98_O_26_	1210.6346	1209.6268	1209.6298	−2.5
14	11.679	ginsenoside F1	C_36_H_62_O_9_	638.4394	637.4315	683.4375 [M−H+HCOOH]^−^	0.9
15	11.703	ginsenoside Rb2	C_53_H_90_O_22_	1078.5923	1077.5845	1077.5885	3.7
16	12.018	ginsenoside Rb3	C_53_H_90_O_22_	1078.5923	1077.5845	1077.5896	4.7
17	14.143	ginsenoside Rd	C_48_H_82_O_18_	946.5501	945.5423	945.5416	−0.7
18	17.424	ginsenoside Rg6	C_42_H_70_O_12_	766.4867	765.4789	765.4766	−3.0
19	17.572	ginsenoside Rk3	C_36_H_60_O_8_	620.4288	619.4195	665.427 [M−H+HCOOH]^−^	1.1
20	17.583	ginsenoside F4	C_42_H_70_O_12_	766.4867	765.4789	765.4700	11.6
21	17.656	ginsenoside F2	C_42_H_72_O_13_	784.4973	783.4894	783.4823	9.1
22	17.680	ginsenoside Rh4	C_36_H_60_O_8_	620.4288	619.4210	665.4254 [M−H+HCOOH]^−^	−1.7
23	17.845	ginsenoside Rg3	C_42_H_72_O_13_	784.4973	783.4894	783.4849	−5.7
24	18.357	ginsenoside Rk1	C_42_H_70_O_12_	766.4867	765.4789	765.4812	3.0
25	18.398	ginsenoside Rg5	C_42_H_70_O_12_	766.4867	765.4789	765.4799	1.3
26	18.436	compound K	C_36_H_62_O_8_	622.4445	621.4366	621.4358	−1.2
27	18.564	ginsenoside Rh2	C_36_H_62_O_8_	622.4445	621.4366	667.4415	−0.9

### 2.3. Prediction of Ginseng Origin with PLS

**Figure 5:**
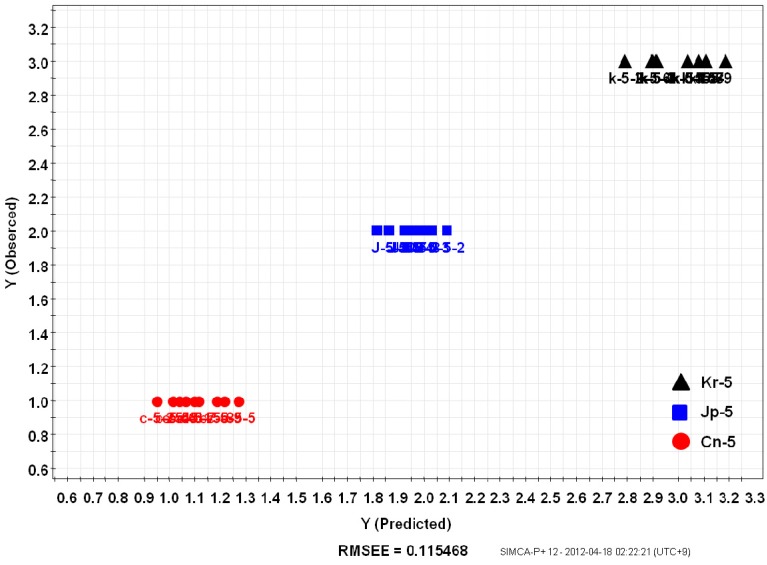
PLS-derived relationship between observed and estimated origin of the 5 year old *Panax ginseng* root samples. Twenty-nine samples were used as a training set (Korea: Kr-5, Japan: Jp-5, China: Cn-5).

**Figure 6:**
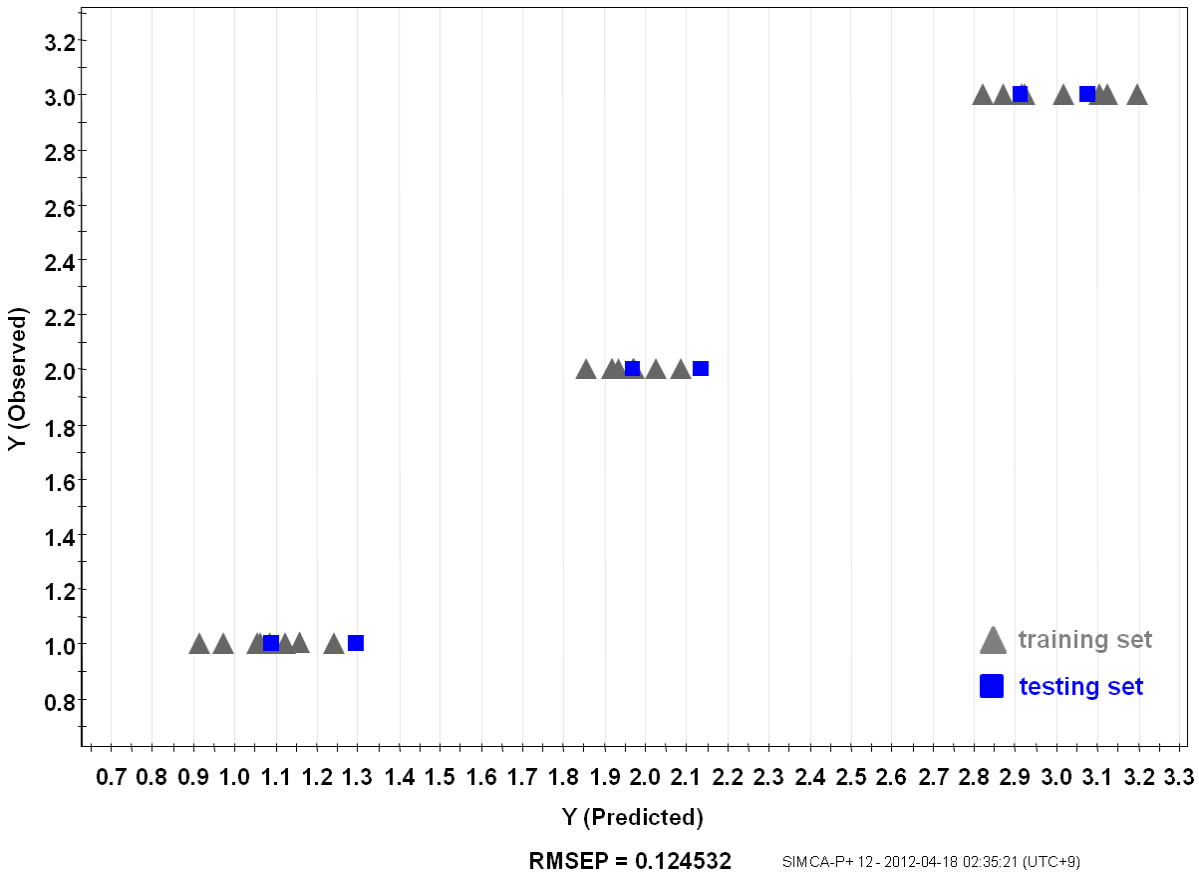
Relationship between observed and predicted *Panax ginseng* origin of PLS model. Training set (gray) and testing set (blue).

PLS, metabolite fingerprinting with projection to latent structure, was considered as an algorithm to create a quality prediction model for ginsengs. A relationship between a sample’s metabolite profiling (X variables) and its origin (Y variables) was observed [11]. Samples were divided into groups of training and testing sets. The PLS-derived relationship between the observed and estimated origin of the Korean (Kr-5), Chinese (Cn-5), and Japanese (Jp-5) *P. ginseng* samples using the total peak area of the training set are shown in Figure 5. Y variables that were original to each sample, were chosen arbitrarily (**1**: Cn-5; **2**: Jp-5; **3**: Kr-5). The training set included individual samples of Kr-5, Cn-5, and Jp-5 ginseng roots, and two random samples were used as a testing set for the model (Figure 6). Prediction after subjecting a test set into the model allowed the predictive accuracy for test samples [Root-Mean-Square Error of Prediction (RMSEP) = 0.115468] to be in a good agreement with the model estimations based on the training samples [Root-Mean-Square Error of Estimation (RMSEE) = 0.124532].

## 3. Experimental

### 3.1. *Panax ginseng* Samples

Five-year-old Korean *P. ginseng* roots were provided by a Punggi ginseng cooperative, Punggi County, Gyeongbuk Province, Korea in 2009. Chinese *P. ginseng* roots cultivated for 5 years were collected locally from three villages (Antu, Dunhua, and Wangcheng) of the Jinlin Province, China in 2009. Five-year-old Japanese *P.* *ginseng* roots were provided by a folk medicine market, Fukushima, Japan, in 2009 (Figure S2). Voucher specimens were deposited in the Natural Products Chemistry Laboratory, Kyung Hee University, Yongin, Korea. Each sample’s number, weight, length, location of collection, and parts are listed in the supplementary information (Table S1).

### 3.2. Standard Constituents and Reagents

HPLC-grade acetonitrile, methanol, and water were obtained from Merck (Darmstadt, Germany). Formic acid was purchased from Sigma-Aldrich (St. Louis, MO, USA). Ginsenosides were isolated and purified from *P. ginseng* roots and red ginseng by a series of chromatography procedures in our laboratory, and their structures were elucidated by a comparison of spectroscopic data (MS, ^1^H-NMR, and ^13^C-NMR) with the literature data: ginsenoside Rb1 [12], Ra2 [13], Ra3 [13], Rc [12], Ra1 [13], Rb2 [12], Rb3 [14], Rd [12], F2 [15], Rg3 [15], Rh2 [16], compound K [15], 20-*O*-glucoginsenoside Rf [14], ginsenoside Rg1 [17], Rg2 [17], Re [17], Rf [18], Rh1 [17], notoginsenoside R2 [17], ginsenoside F1 [19], Rg6 [20], Rk1 [21], Ro [22], and Rk3 [21], ginsenoside F4 [23], Rh4 [17], and Rg5 [24]. The purity of the isolated compounds was determined to be more than 98% by normalization of the peak areas detected by HPLC analysis. The chemical structures of the investigated ginsenosides are shown in Table 2.

**Table 2:** Structures of ginsenosides (Glc: *β*-d-glucopyranosyl, Ara(p): *α*-l-arabinopyranosyl, Ara(f): *α*-l-arabinofuranosyl, Rha: *α*-l-rhamnopyranosyl, Xyl: *β*-d-xylopyranosyl, glcU: *β*-d-glucuronic acid).

Chemical structures	Saponins	R_1_	R_2_
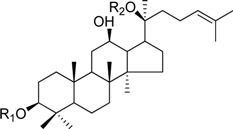	ginsenoside Rb1	-Glc^2^-Glc	-Glc^6^-Glc
	ginsenoside Ra2	-Glc^2^-Glc	-Glc^6^-Ara(f)^2^-Xyl
	ginsenoside Ra3	-Glc^2^-Glc	-Glc^6^-Glc^3^-Xyl
	ginsenoside Rc	-Glc^2^-Glc	-Glc^6^-Ara(p)
	ginsenoside Ra1	-Glc^2^-Glc	-Glc^6^-Ara(p)^4^-Xyl
	ginsenoside Rb2	-Glc^2^-Glc	-Glc^6^-Ara(f)
	ginsenoside Rb3	-Glc^2^-Glc	-Glc^6^-Xyl
	ginsenoside Rd	-Glc^2^-Glc	-Glc
	ginsenoside F2	-Glc	-Glc
	ginsenoside Rg3	-Glc^2^-Glc	-Glc^6^-Glc
	compound K	-H	-Glc
	ginsenoside Rh2	-Glc	-H
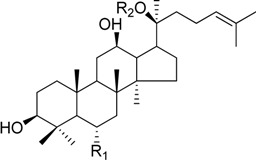	20-*O*-gluco-ginsenoside Rf	-Glc^2^-Glc	-Glc
	ginsenoside Rg1	-Glc	-Glc
	ginsenoside Rg2	-Glc^2^-Rha	-H
	ginsenoside Re	-Glc^2^-Rha	-Glc
	ginsenoside Rf	-Glc^2^-Glc	-OH
	ginsenoside Rh1	-Glc	-H
	notoginsenoside R2	-Glc^2^-Xyl	-H
	ginsenoside F1	-OH	-Glc
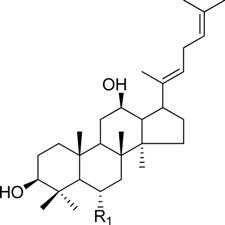	ginsenoside Rg6	-Glc^2^-Rha	
	ginsenoside Rk3	-Glc	
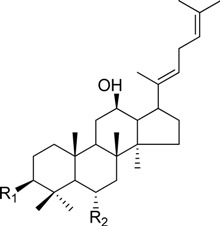	ginsenoside F4	-H	-Glc^2^-Rha
	ginsenoside Rh4	-H	-Glc
	ginsenoside Rg5	-Glc^2^-Glc	-H
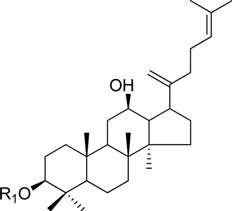	Ginsenoside Rk1	-Glc^2^-Glc
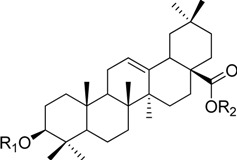	Ginsenoside Ro	-GlcU^2^-Glc	-Glc

### 3.3. Sample Preparation

Each sample was dried at 40 °C in a forced air convection drying oven for 48 h after washing, and then weighed. The main and lateral roots were used for experiments after removing rhizomes and fine roots. Roots were ground (<0.5 mm) using a mixer (Hanil, Seoul, Korea) and thoroughly mixed, and subsamples were homogenized further using a Retsch MM400 mixer mill (Retsch GmbH, Haan, Germany) for analyses. Fine powder was weighed (100 mg), suspended in 1.0 mL of 70% (v/v) methanol, and ultrasonically extracted for 1 h at 50 °C. The extract was then evaporated under reduced pressure, and the residue was dissolved in 70% methanol. The solution was filtered through a syringe filter (0.22 µm) and injected directly into the RRLC system. A blank sample (3 µL) consisting of 70% methanol was injected between selected analyses to validate inter-sample cross-talking effects.

### 3.4. RRLC-QTOF-MS Analysis

RRLC was performed on a 1200 RRLC SL+ system (Agilent, Santa Clara, CA, USA) equipped with a binary solvent delivery system and an auto-sampler. Chromatographic separations were performed on a 2.1 × 100 mm, 1.8 µm RRHT C_18_ chromatography column. The column oven was maintained at 40 °C and the mobile phases consisted of solvent A (0.1% formic acid in water, v/v) and solvent B (0.1% formic acid in acetonitrile, v/v). Optimized LC elution conditions were as follows: 0–1 min, 25% B; 1–5 min, 30% B; 5–15 min, 75% B; 15–18 min, 90% B; 18–20 min, 100% B and 20–23 min, 25% B. The flow rate was 0.60 mL/min. A 3 µL aliquot was injected onto the column using the auto-sampler. MS analysis was performed using a 6530 QTOF-MS (Agilent) operating in the negative ion electrospray mode. The operating parameters were: drying gas (N_2_) flow rate, 5.0 L/min; drying gas temperature, 350 °C; nebulizer, 45 psig; sheath gas temperature, 400 °C; sheath gas flow, 12 L/min; capillary, 3500 V; skimmer, 65 V; OCT RF V, 750 V; and fragmentor voltage, 175 V. For MS/MS experiments, the collision energy was adjusted from 20 V to 50 V to optimize signals and obtain maximal structural information from the ions of interest. The data scan time was set to 0.5 s with a 0.02 s interscan delay. The number of scans to sum automatically defaults to 2. Set the number of cycles to 5. This will result in a total of 10 scans performed. The mass range was set at *m/z* 100–1500.

### 3.5. Data Preprocessing

Data processing was performed on both Mass Hunter workstation (software version B.02.04 provided by Agilent, USA) and MZmine [25]. With Mass Hunter, each mass number was analyzed separately in a search peak. The area of the peak was given an identity of *m*/*z* and a retention time, which were used as fingerprints. For MZmine, raw chromatographic data were converted into NetCDF format (.cdf) before applying to the software. Peak detection finds the peaks corresponding to the compounds. Alignment aims at matching the corresponding peaks across multiple sample runs. Spectral filtering aims at reducing the complexity of data and removing the noise. The role of normalization is to reduce the systematic error by adjusting the intensities within each sample run.

### 3.6. Multivariate Analysis

Principal component analysis (PCA), using SIMCA-P^+^ version 12.0 (Umetrics, Umeå, Sweden), was initially executed to understand the relationship expressed in terms of similarities or dissimilarities among groups of multivariate data. Projections to latent structures by means of partial least-squares (PLS), using SIMCA-P^+^ version 12.0, were subsequently performed to create a quality-prediction model.

## 4. Conclusions

RRLC-QTOF/MS, which was developed in combination with metabolomics, provides a candidate group or marker with a fast, reliable, and precise quality assessment method. By observing PCA plots, the ginseng samples were able to be aligned with respect to the location in which the roots were cultivated. The roots from Korea, China, and Japan were aligned accordingly and indicated that variations in the metabolite fingerprints from the different samples are geographically influenced; leading to geographically influenced distinctive classifications, even within these 5-year old *P. ginseng* root samples. Our results indicate that cultivation region is a major influential factor for quality discrimination. A technique such a RRLC-QTOF/MS-based metabolite profiling is promising for revealing and elucidating the metabolic outcomes as a result of geographical and seasonal variations, as well as of the different cultivation methods employed. Hence, this work is of great importance for the evaluation of overall quality of medicinal plants, which is ultimately of great significance in the pharmacological and clinical investigation of drug products of plant origin.

## Supplementary Materials

Supplementary materials relating to this article are available online and include descriptions of the *Panax ginseng* root samples (Table S1), representative appearances of *Panax ginseng* roots collected from different countries (Figure S1) and total ion chromatograms (TIC) of ginsenosides from QTOF/MS analysis in the range of 100–1,500 *m/z* (Figures S2). Supplementary materials can be accessed at: http://www.mdpi.com/1420-3049/18/12/14849/s1.
