# Supplementary material for: Quality Evaluation of Panax ginseng Roots Using a Rapid Resolution LC-QTOF/MS-Based Metabolomics Approach
Source: Molecules. 2013 Dec 3;18(12):14849–61. doi: 10.3390/molecules181214849 (PMC6269782; doi:10.3390/molecules181214849)
Supplement: Supplementary file 1 [file molecules-18-14849-s001.pdf]

Supporting Information

Figure S1. TIC of ginsenosides from QTOF/MS analysis in the range of 100–1,500 *m/z*.

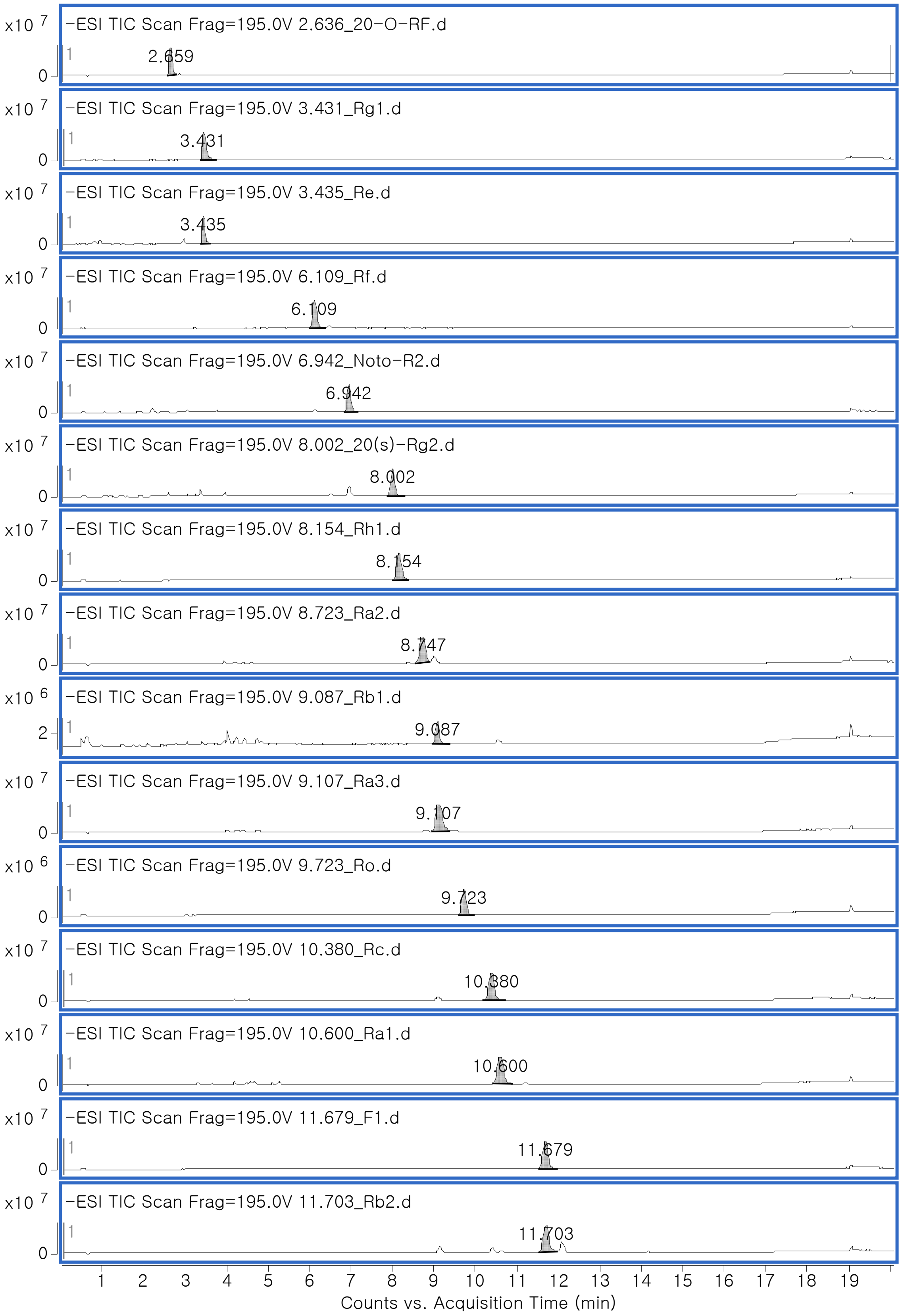

Figure S1. *Cont.*

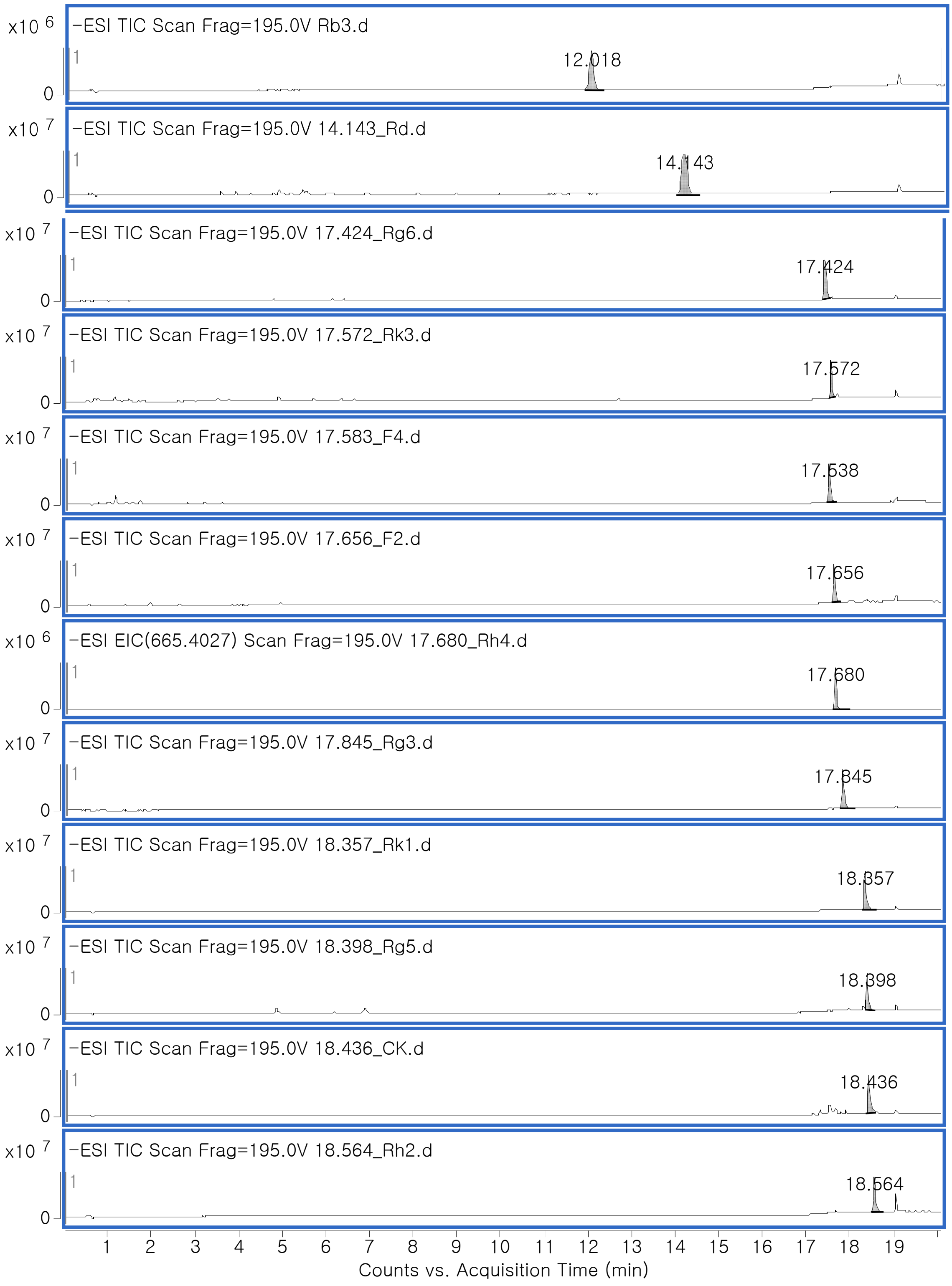

Figure S2. Representative appearances of *Panax ginseng* roots collected from different countries.

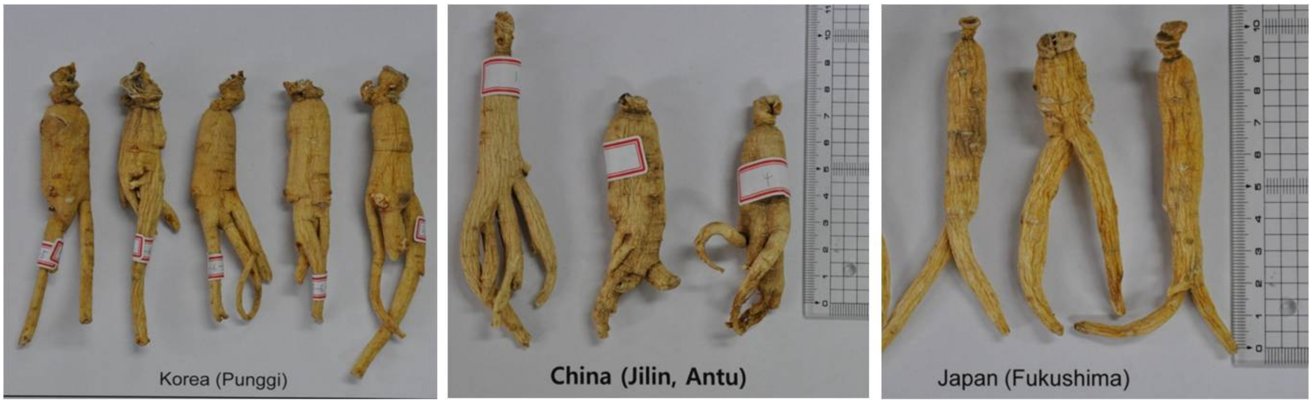

Table S1. Description of *Panax ginseng* root samples (Kr: Korea, Cn: China, Jp: Japan).

Sample No.	Weigh (g)	Main root diameter (cm)	Main root length (cm)	Lateral roots (ea)	Location
Kr-5-1	30.9	2.2	9.3	3	Sunheung
Kr-5-2	26.8	2	10.7	2	
Kr-5-3	33.8	2.3	11.3	1	
Kr-5-4	30.5	2.2	8.2	3	Anjeong
Kr-5-5	20.9	2.1	6.5	2	
Kr-5-6	33.9	2.3	11.8	2	
Kr-5-7	26	1.9	7.1	5	Sambup
Kr-5-8	33.2	2.6	5	6	
Kr-5-9	36.4	2.3	9	3	
Kr-5-10	27.9	2.5	1.5	6	
Cn-5-1	16.3	3.5	1.5	5	Antu
Cn-5-2	16	3.5	2.2	3	
Cn-5-3	14.1	3.9	2.1	3	
Cn-5-4	25.4	6.5	2	3	Dunhua
Cn-5-5	25.5	6.5	2.1	2	
Cn-5-6	23.1	6.4	2	5	
Cn-5-7	22	6.3	2	2	Wangcheng
Cn-5-8	19.6	6.5	2.3	2	
Cn-5-9	19.4	6.7	1.7	3	
Jp-1	9.7	1.5	4	2	Fukusima
Jp-2	7.6	1.5	4.7	2	
Jp-3	7.7	1.4	6.7	1	
Jp-4	8.3	1.8	2.5	2	
Jp-5	8.1	1.2	5.5	2	
Jp-6	6.9	1.5	4.7	2	
Jp-7	6.1	1.4	3.4	2	
Jp-8	11.7	1.6	6.3	2	
Jp-9	7.2	1.2	8.2	2	
Jp-10	11.4	1.4	5.8	1	
